# The Link Between Mate Value Discrepancy and Relationship Satisfaction—An Empirical Examination Using Response Surface Analysis

**DOI:** 10.3390/bs15081131

**Published:** 2025-08-20

**Authors:** Mehmet Mehmetoglu, Ilmari Määttänen, Matthias Mittner

**Affiliations:** 1 Department of Psychology, Norwegian University of Science and Technology, Edvards Bull veg 1, 7491 Trondheim, Norway; matthias.mittner@uit.no; 2 Department of Psychology, University of Helsinki, Haartmaninkatu 3, 00014 Helsinki, Finland; ilmari.maattanen@helsinki.fi; 3 Institute for Psychology, UiT—The Arctic University of Norway, Huginbakken 32, 9037 Tromsø, Norway

**Keywords:** mate value, relationship, satisfaction, couple, romantic, evolutionary, psychology

## Abstract

Existing studies on mate value discrepancy and relationship satisfaction often suffer from two key limitations: they conceptualize mate value as a single, undifferentiated construct and rely on simple difference scores to model discrepancy effects. The present study addresses these issues by examining the relationship between mate value discrepancy and relationship satisfaction using a multidimensional operationalization of mate value and applying response surface analysis. Data were collected in 2016 in Norway via a web-based survey administered by a market research company, with a sample stratified across the country’s 19 counties. The final estimation sample included 904 individuals currently in romantic relationships. The analysis involved a combination of confirmatory factor analysis and response surface analysis. The findings indicate that relationship satisfaction is highest when both partners exhibit high levels of Family orientation, resourcefulness, appearance, sociability, and physical condition. Among these, family orientation emerged as the most important dimension. Notably, relationship satisfaction declined when both partners scored equally low on these traits. Implications for future research and theoretical implications are discussed.

## 1. Introduction

Humans are generally a culturally monogamous species, forming long-lasting pair bonds. Most human cultures predominantly practice monogamy, and monogamous marriage has been the norm in all major modern societies (Schacht & Kramer, 2019). However, humans are not strictly monogamous; it is common for individuals to change partners multiple times throughout their lives or even to have multiple partners concurrently. This flexibility in human mating patterns influences mate choice, mate acquisition, and mate retention strategies. The “mate switching hypothesis” (Buss et al., 2017) offers insight into infidelity as a potential strategy. According to this hypothesis, individuals may maintain “back-up” mates, evaluate alternative mating opportunities, weigh the associated risks and costs, and assess discrepancies in mate value with their current partner.

Relationship satisfaction plays a key role in important outcomes, such as the stability of monogamous relationships. Different aspects of monogamous mating—such as attracting and retaining a partner—may sometimes conflict. From an individual’s perspective, it is desirable to secure a partner of high “mate value” (MV). Mate value refers to the total potential fitness benefits a prospective partner can provide, encompassing phenotypic condition, genetic quality, and resource holding (Howie & Pomiankowski, 2021). Individuals are expected to evolve preferences that maximize their partner’s mate value, which (being difficult to assess directly) is inferred from physical and behavioral traits shaped by the selective pressures of mate choice (Howie & Pomiankowski, 2021). However, choosing a partner with a significantly higher mate value than oneself may create challenges for relationship stability. For example, greater mate value discrepancy, where one partner has a higher mate value, has been linked to increased digital dating abuse (Bhogal et al., 2019; Singh Bhogal & Howman, 2019) and a greater likelihood of forgiving infidelity (Sidelinger & Booth-Butterfield, 2007). Research has shown that controlling behaviors are most intense in couples where the woman perceives her mate value as higher than that of her partner (Danel et al., 2017). Additionally, individuals who view themselves as having a higher mate value than their partners report engaging in more cost-inflicting behaviors and also experiencing such behaviors from their partners (Young et al., 2024). Therefore, mate value discrepancy between partners is a significant factor influencing relationship satisfaction, which in turn affects the quality and stability of the relationship (Danel et al., 2017).

Relationship satisfaction has been linked to the relative desirability of one’s mate compared to alternative partners, but this effect is most pronounced in individuals who perceive their own mate value as higher than that of their partner (Conroy-Beam et al., 2016). Interestingly, individuals tend to be more sensitive to negative discrepancies—where their partner’s qualities fall below ideal standards—than to positive ones. In other words, having a partner who exceeds ideal standards does not necessarily enhance relationship satisfaction (Buyukcan-Tetik et al., 2017).

Previous studies on relationship satisfaction and mate value discrepancies have typically focused on a single, generalized “mate value” variable rather than examining specific mate value traits separately. Findings indicate that when a partner’s perceived mate value is lower than one’s own, relationship satisfaction tends to decrease if potential alternative partners are seen as more desirable than the current partner (Conroy-Beam et al., 2016), suggesting an influence of partner substitutability. However, the distinct roles of different mate value traits remain unclear, making it challenging to form precise hypotheses. For example, obtaining a partner who is both physically attractive and wealthy may be more difficult than securing one with other desirable traits. In such cases, lower partner attractiveness or resources compared to oneself might predict lower relationship satisfaction. Supporting this, Starratt et al. (2017) found that a partner’s physical attractiveness is the strongest predictor of reduced sexual infidelity in males, suggesting that physical attractiveness serves as a protective factor in relationships.

Homogamy, the preference for similar mates to oneself, is well-documented in psychological and physical traits (Thiessen & Gregg, 1980). Direct homogamy is not the only source of mate similarity. Mate similarity may result from sexual imprinting from the same-sex parent, influencing personality (Gyuris et al., 2010), body shape (Štěrbová et al., 2018), and ideal mate traits like eye and hair color (Štěrbová et al., 2019). Thus, partner similarity can arise from various paths, including social learning from parents. In this study, the “resourcefulness” preference aligns with typical socioeconomic and educational homogamy (Mare, 2016). The “attractiveness” preference in this study evaluates how well individual preferences are met without detailing specific traits.

In light of the reviewed literature, the aim of this study is to examine the link between mate value discrepancy (the difference between a person’s perceived own mate value and her/his partner’s mate value) and relationship satisfaction. We also refer to the former as “self” and the latter as “partner” where necessary in the paper. There already exist some studies that have inquired into how mate value discrepancy may affect relationship satisfaction and other related outcome variables (e.g., relationship length). Although these studies have successfully informed us about the intriguing link between mate value and relationship satisfaction, they still suffer mainly from two limitations.

The first limitation is that, in these studies, mate value has been operationalized as a single omnibus construct rather than as a multi-faceted phenomenon. The second limitation is a methodological one, in that in many of these studies, the relationship satisfaction variable is regressed directly on a difference score variable. However, that approach discards potentially important information about the reference levels of the original scores on which the mismatches (self ≠ partner) occur. For example, a difference of self-partner =1 could occur for self =2 and partner =1, or the same mismatch could occur for self =4 and partner =3, and both situations may potentially result in different outcomes. The same applies to matches (i.e., self = partner), which can have different effects depending on the values of “self” and “partner”.

To overcome the first limitation, we operationalize mate value as consisting of multiple dimensions (family-orientation, resourcefulness, appearance, sociability, and physical condition). The second limitation is adequately addressed by employing the so-called response surface analysis (RSA), facilitated by the RSA package (Schönbrodt & Humberg, 2023). RSA can further provide us with an estimate of the effect of the matched cases (in addition to the mismatched cases) on the outcome variable, which is largely neglected in the literature. There are additional advantages of using RSA over the difference score method, including greater statistical power, flexibility and the ability to model assymetric patterns, which are treated in detail elsewhere in the literature (Barranti et al., 2017; Shanock et al., 2010).

## 2. Methods

### 2.1. Sample

The data for this study were collected in 2016 in Norway via a web survey administered by a market research company. The sample was stratified according to the population distribution across Norway’s 19 counties. As shown in Table 1, the final estimation sample comprised 904 individuals who were currently in a romantic relationship. Approximately 10% of participants had been in their relationship for less than 4 years, 14% for 4 to 10 years, and 76% for more than 10 years. The sample included 488 men and 416 women, with an age range of 18 to 86 years (*M* = 51.87, SD=15.76).

Regarding household composition, nearly 70% of respondents reported having no children under the age of 18 living in their household, whereas 30% indicated they had at least one child under 18. The sample was relatively highly educated: nearly 64% were either currently pursuing a bachelor’s degree or had already obtained a bachelor’s or higher degree, while the remaining 33% had a secondary or high school education.

### 2.2. Measures

In line with the aim of the study, we defined relationship satisfaction as the dependent variable and mate value as the independent variables. Relationship satisfaction was measured using the 7-item scale developed by Hendrick (1988). The respondents were asked to indicate on a 5-point scale (1 = little degree and 5 = large degree) to what degree they felt that “they loved their partners”, “they were satisfied with their relationships”, and so on (see Table 2). The study further adopted the mate value scale proposed by Kirsner et al. (2003). Our adjusted list (see Table 2), however, included 20 items. The respondents were asked to indicate how well each of the initial 20 mate preference criteria (e.g., understanding, socially intelligent, humorous, and successful) described them as well as their partner as a person. These items used a 5-point scale ranging from 1 (very poorly) to 5 (very well).

### 2.3. Data Analysis

All 904 respondents completed the entire questionnaire with no missing data. The data analysis consisted of two sequential parts: a measurement part followed by a structural one. The measurement part addresses the relationships between latent and observed variables (i.e., mate value, relationship satisfaction, and their respective items), whereas the structural part concerns hypothesis testing between latent variables—in particular, the relationship between the independent variables (i.e., mate value) and the dependent variable (relationship satisfaction).

#### 2.3.1. Measurement Part

The measurement part comprised a confirmatory factor analysis (CFA) estimated using the lavaan package in R (Rosseel, 2012). The CFA model included the same five latent variables (10 altogether), representing both the respondent’s perceived own mate value and their partner’s perceived mate value, using the same 20 items for each (40 altogether). As we expected self and partner mate value evaluations to be correlated, we correlated their residuals in our CFA model. Finally, we added relationship satisfaction as a single latent variable together with its respective items to the CFA model. The CFA model exhibited acceptable fit measures: RMSEA (root mean square error of approximation) was 0.055, CFI (comparative fit index) was 0.894, and TLI (Tucker–Lewis index) was 0.881. More importantly, the latent variables were found to have convergent validity (the latent variable explains enough variance in the items) and discriminant validity (latent variables are distinct from each other). Furthermore, the standardized loadings were all satisfactory (significant and substantial) as displayed in Table 2.

#### 2.3.2. Structural Part

The structural part of the model involved a response surface analysis (RSA) using the RSA package (Schönbrodt & Humberg, 2023) in R. Since the RSA package can only accommodate observed variables, we created average scores for each of the latent variables representing the five dimensions of mate value and relationship satisfaction based on their respective items. Subsequently, we also computed the reliability coefficients for all scales, all of which proved to be satisfactory (see Table 2). Finally, we performed the main analysis, namely RSA, which conceptually consists of two steps: (a) running a polynomial regression model, and (b) using the coefficients from this model to generate a response surface plot and test whether, and in what way, matches and mismatches between self- and partner’s mate value matter (Barranti et al., 2017). As our presentation of response surface analysis (RSA) from this point onward may appear rather technical, readers seeking a more intuitive and detailed explanation of the method may refer to Barranti et al. (2017). The polynomial regression model is given by

(1) $$E\left[Z_{i}\right]=\beta_{0}+\beta_{1}X_{i}+\beta_{2}Y_{i}+\beta_{3}X_{i}^{2}+\beta_{4}\left(X_{i}\times Y_{i}\right)+\beta_{5}Y_{i}^{2}$$

where *X* represents the respondent’s score on each of the mate value dimensions (e.g., Family orientation, resourcefulness, appearance, sociability, and physical condition), and *Y* represents the score of the partner of the respondent on the same dimension. The polynomial regression model includes both variables as predictors, as well as their squared terms ($X^{2}$ and $Y^{2}$) and their interaction term (X×Y). E[Z] refers to the expected value of *Z* (relationship satisfaction) as predicted by the model. The model is estimated using centered *X* and *Y* variables. Centering both predictors at the midpoint of the scale (3) ensures interpretability of the regression coefficients (Barranti et al., 2017).

**Figure 1:**
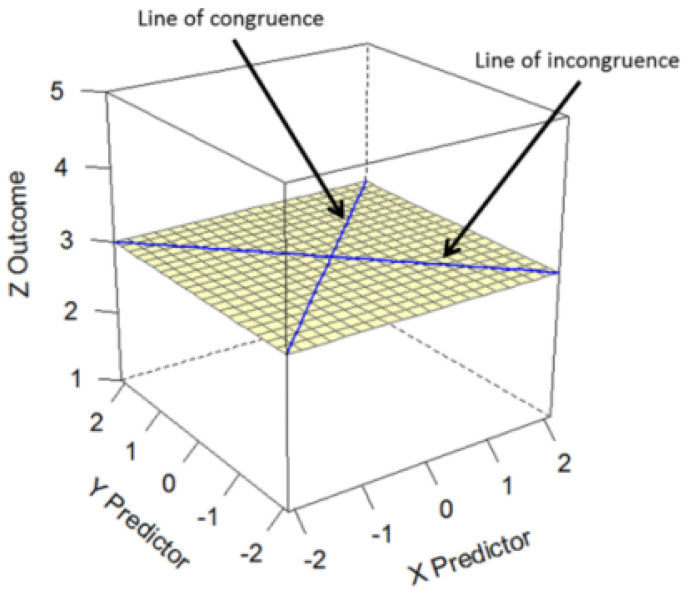
Response surface. Source: Barranti et al. (2017).

In the second step, Equation (1) is evaluated at a grid of (X,Y) pairs using the estimated parameter values, producing the so-called “response surface”—the model predictions of the polynomial regression model for all pairs of predictor values. The RSA package automatically produces such a response surface, an example of which is shown in Figure 1. As shown in Figure 1, the *X* (self) and *Y* (partner) axes range from negative to positive values, with 0 reflecting the midpoint of the scale. The *Z* axis shows the outcome on its original metric. For instance, the top right corner (TRC) depicts the predicted mean *Z* value when both *X* (+2) and *Y* (+2) are maximal, whereas the top left corner (TLC) shows the predicted mean *Z* value when *X* (–2) is at its lowest and *Y* (+2) is maximal. Moreover, while the bottom right corner (BRC) shows the predicted mean *Z* value when *X* (+2) is the highest and *Y* (–2) is the lowest, the bottom left corner (BLC) shows the predicted mean *Z* value when *X* (–2) is the lowest and *Y* (+2) is the highest.

Figure 1 shows two additional lines. The line running from BLC to TRC is the line of congruence (LOC), reflecting cases where the values of *X* and *Y* perfectly match at all levels of the scale (i.e., where the assessments of self and partner mate value coincide). In contrast, the line running from BRC to TLC is the line of incongruence (LOIC), representing cases where the values of *X* are the opposite of those of *Y*. Through linear combinations of the coefficients from Equation (1), we can further estimate the so-called surface test values (see Equations (2)–(5)), provided that the R-squared values are significantly different from zero (Shanock et al., 2010), which was the case in the current analysis:

(2) $$\begin{array}{cc} a_{1} & =\beta_{1}+\beta_{2} \end{array}$$

(3) $$\begin{array}{cc} a_{2} & =\beta_{3}+\beta_{4}+\beta_{5} \end{array}$$

(4) $$\begin{array}{cc} a_{3} & =\beta_{1}-\beta_{2} \end{array}$$

(5) $$\begin{array}{cc} a_{4} & =\beta_{3}-\beta_{4}+\beta_{5} \end{array}$$

**Figure 2:**
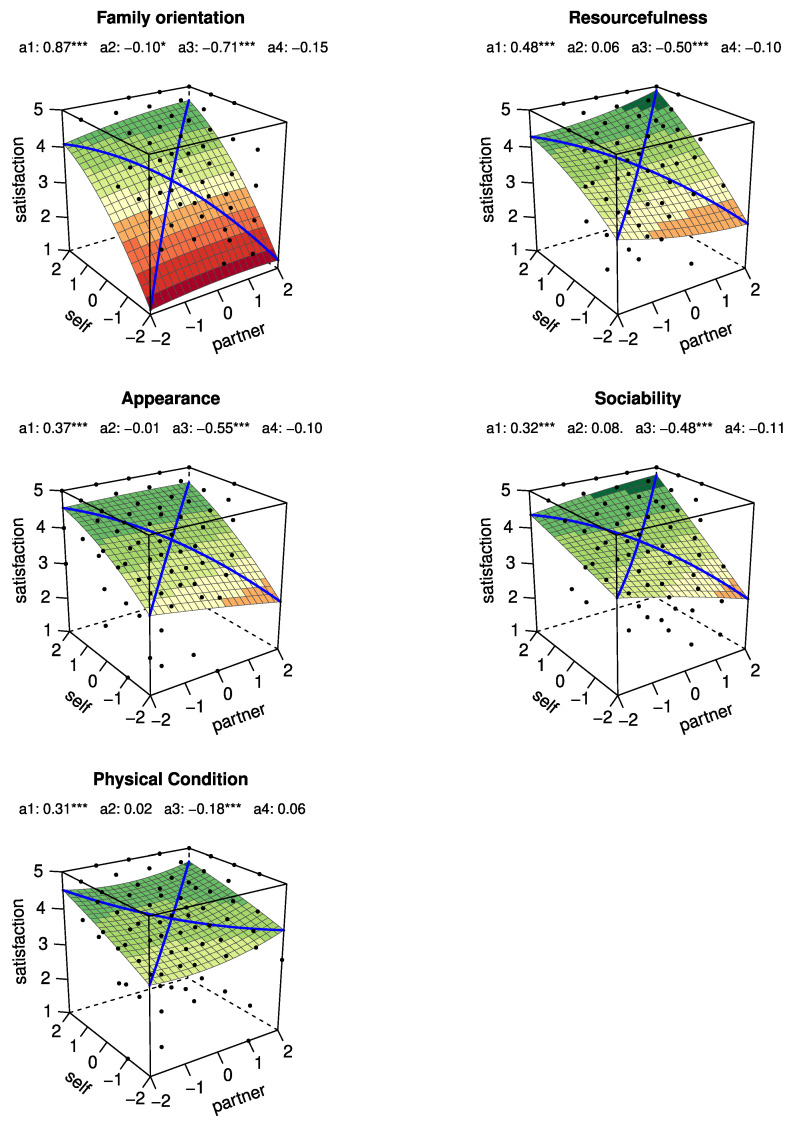
Response surface plots for the link between a person’s perceived self and her/his partner’s mate value on relationship satisfaction. * = p<0.05, *** = p<0.001.

More specifically, $a_{1}$ can be used to test the slope of the LOC, and $a_{2}$ tests the curvature of the LOC, whereas $a_{3}$ tests the slope of the LOIC and $a_{4}$ tests the curvature of the LOIC. These surface test values are automatically computed and provided on the default 3D plots generated by the RSA package (see Figure 2). When conducting an RSA, we typically use these figures (TRC, TLC, BRC, and BLC) and surface test values ($a_{1}$, $a_{2}$, $a_{3}$, and $a_{4}$) to interpret the results of the polynomial regressions and to draw conclusions about how matches and mismatches between self and partner mate value relate to relationship satisfaction.

## 3. Results and Findings

In what follows, we present the association between mate value discrepancy or similarity on each of the mate value criteria (family-orientation, resourcefulness, appearance, sociability, and physical condition) and relationship satisfaction.

### 3.1. Family Orientation

As we can see in Figure 2, when inspecting the line of congruence (i.e., self = partner), as we move from lower to higher levels of Family orientation (for both self and partner), relationship satisfaction increases ($a_{1}=0.87,\;p<0.001$). However, this increase is a nonlinear one ($a_{2}=-0.10,\;p<0.05$), suggesting that relationship satisfaction increases less sharply for similarity at the highest versus lowest levels of family orientation. Relatedly, TRC (i.e., self =2 and partner =2) and BLC (i.e., self =−2 and partner =−2) are associated with the scores of 4.60 and 1.25 on relationship satisfaction, respectively. Moreover, as we move from the scenario of self > partner to partner > self on Family orientation, relationship satisfaction increases and vice versa ($a_{3}=-0.71,\;p<0.001$). This association appears to be a linear one ($a_{4}=-0.15,\;n.s.$) suggesting further that in both scenarios (self > partner and partner > self), relationship satisfaction increases or decreases at the same rate. As such, BRC (i.e., self =2 and partner =−2) and TLC (i.e., self =−2 and partner =+2) are linked with the scores of 1.24 and 4.09 on relationship satisfaction, respectively.

### 3.2. Resourcefulness

Again, as can be observed from Figure 2, as we move from lower levels of similarity (i.e., self = partner) to higher levels of similarity on resourcefulness, relationship satisfaction increases ($a_{1}=0.48,\;p<0.001$). This increase follows a linear trend ($a_{2}=0.06,\;n.s.$) indicating that relationship satisfaction increases at the same rate for similarity at the highest versus lowest levels of resourcefulness. It follows then that TRC (i.e., self =2 and partner =2) and BLC (i.e., self =−2 and partner =−2) are associated with the scores of 4.90 and 2.96 on relationship satisfaction, respectively. Furthermore, as we move from the scenario of self > partner to partner > self on resourcefulness, relationship satisfaction increases and vice versa ($a_{3}=-0.50,\;p<0.001$). This association appears to be a linear one too ($a_{4}=-0.10,\;n.s.$), indicating further that in both scenarios relationship satisfaction increases or decreases at the same rate. Moreover, BRC (i.e., self =2 and partner =−2) and TLC (i.e., self =−2 and partner =+2) are linked with the scores of 2.28 and 4.29 on relationship satisfaction, respectively.

### 3.3. Appearance

As depicted in Figure 2, as we move from lower levels of similarity (i.e., self = partner) to higher levels of similarity on appearance, relationship satisfaction increases ($a_{1}\;=\;0.37$, p<0.001). This increase is a linear one ($a_{2}=-0.01,\;n.s.$), telling us that relationship satisfaction increases at the same rate for similarity at the highest versus lowest levels of appearance. TRC (i.e., self =2 and partner =2) and BLC (i.e., self =−2 and partner =−2) are accordingly associated with the scores of 4.56 and 3.08 on relationship satisfaction, respectively. Moreover, as we move from the scenario of self > partner to partner > self on appearance, relationship satisfaction increases and vice versa ($a_{3}=-0.55,\;p<0.001$). This association appears to be a linear one too ($a_{4}=-0.10,\;n.s.$), suggesting further that in both scenarios relationship satisfaction increases or decreases at the same rate. Moreover, BRC (i.e., self =2 and partner =−2) and TLC (i.e., self =−2 and partner =+2) are linked with the scores of 2.36 and 4.55 on relationship satisfaction, respectively.

### 3.4. Sociability

As shown in Figure 2, as we move from lower levels of similarity (i.e., self = partner) to higher levels of similarity on sociability, relationship satisfaction increases ($a_{1}=0.32,\;p<0.001$). This increase follows a linear pattern ($a_{2}=0.08,\;n.s.$) suggesting that relationship satisfaction increases at the same rate for similarity at the highest versus lowest levels of sociability. TRC (i.e., self =2 and partner =2) and BLC (i.e., self =−2 and partner =−2) are thus associated with the scores of 4.78 and 3.51 on relationship satisfaction, respectively. As we move from the scenario of self > partner to partner > self on sociability, relationship satisfaction increases and vice versa ($a_{3}=-0.48,\;p<0.001$). This link seems to be a linear one too ($a_{4}=-0.11,\;n.s.$), indicating further that in both scenarios relationship satisfaction increases or decreases at the same rate. Moreover, BRC (i.e., self =2 and partner =−2) and TLC (i.e., self =−2 and partner =+2) are linked with the scores of 2.44 and 4.36 on relationship satisfaction, respectively.

### 3.5. Physical Condition

As can be observed from Figure 2, as we move from lower levels of similarity (i.e., self = partner) to higher levels of similarity on physical condition, relationship satisfaction increases ($a_{1}=0.31,\;p<0.001$). This increase is a linear one ($a_{2}=0.02,\;n.s.$), implying that relationship satisfaction increases at the same rate for similarity at the highest versus lowest levels of physical condition. TRC (i.e., self =2 and partner =2) and BLC (i.e., self =−2 and partner =−2) are relatedly associated with the scores of 4.62 and 3.36 on relationship satisfaction respectively. As we move from the scenario of self > partner to partner > self on physical condition, relationship satisfaction increases and vice versa ($a_{3}=-0.18,\;p<0.001$). This association seems to be a linear one too ($a_{4}=0.06,\;n.s.$), indicating further that in both scenarios relationship satisfaction increases or decreases at the same rate. Moreover, BRC (i.e., self =2 and partner =−2) and TLC (i.e., self =−2 and partner =+2) are linked with the scores of 3.80 and 4.52 on relationship satisfaction, respectively.

### 3.6. Combined Overview

**Figure 3:**
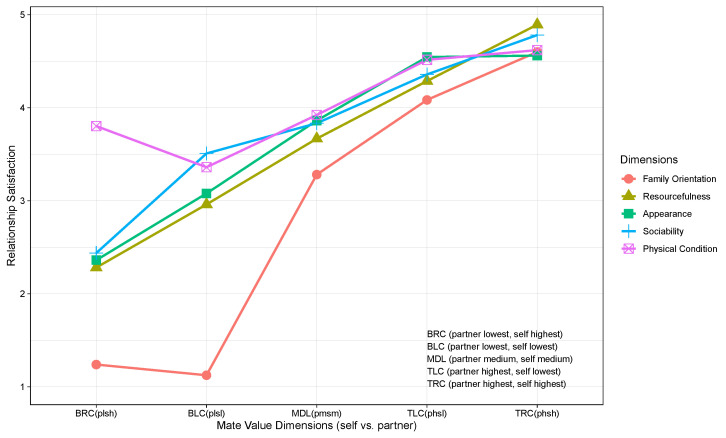
An overview of the main match/mismatch combinations from Figure 1. Y-axis refers to the predicted relationship satisfaction values, whereas the X-axis shows BRC, BLC, TLC, and TRC scenarios as well as MDL (self = 0 and partner = 0). p = partner, s = self, l = lowest, and h = highest.

In Figure 3, we put together all four main combinations of discrepancy and similarity values (BRC, BLC, TLC, and TRC) on all five mate value dimensions (family orientation, resourcefulness, appearance, sociability, and physical condition) for a descriptive analysis. This figure shows that for all the five mate value dimensions, the best scenario (i.e., highest relationship satisfaction) is associated with TRC, which is characterized by a person rating both herself/himself and her/his partner highest on each of the five mate value dimensions. TRC is followed closely by TLC, in which a person rates her/his partner highest and herself/himself lowest on each of the five mate value dimensions. The worst scenario (i.e., lowest relationship satisfaction) is, however, BRC, in which a person rates her/his partner lowest and herself/himself highest on each of the five mate value dimensions.

When we compare the mate value dimensions with each other, we observe an interesting finding: significantly lower relationship satisfaction is observed in the BRC and BLC scenarios for family orientation. This briefly implies that when a partner is rated lowest on family orientation by a person, her/his relationship satisfaction is also lowest—regardless of the person’s own rating. Another finding is related to the mate value dimension of physical condition, in that a moderate level of relationship satisfaction is observed even when a person rates her/his partner lowest on this dimension.

## 4. Discussion and Conclusions

A striking overall finding from the current study is that across all dimensions of mate value (family orientation, resourcefulness, appearance, sociability, and physical condition) individuals tend to prefer both themselves and their partners to possess equally high levels of these traits. This alignment is associated with greater relationship satisfaction. For instance, when both partners are highly family oriented, the relationship tends to benefit. The same pattern holds across the other mate value dimensions.

This preference may reflect several underlying mechanisms, particularly within the Norwegian context. First, it suggests a desire for mutual biological and social investment in offspring, especially with regard to tangible resources and adaptive traits. Second, when both partners are perceived as highly desirable across multiple domains, the relationship may be more balanced, reducing the likelihood of perceived inferiority or asymmetry. Such balance may help prevent psychological tension or conflict that could otherwise arise from discrepancies in mate value.

However, the study’s findings also indicate that when both partners score equally low across the five mate value traits, relationship satisfaction tends to be low. This challenges the commonly held similarity-attraction hypothesis, which posits that individuals are attracted to those similar to themselves. While similarity may still play a role in other contexts, these results suggest that similarity in low mate value does not benefit the relationship and may, in fact, be detrimental. From an evolutionary perspective, this is understandable: two individuals with low mate value are likely to offer limited advantages for offspring, particularly in terms of resource accrual, social desirability, or genetic fitness.

This pattern is especially pronounced for family orientation: when both partners score low on this trait, relationship satisfaction declines most steeply. Family orientation encompasses qualities such as kindness, loyalty, and a strong commitment to family life and shared responsibilities. When both partners lack these qualities, the relationship may suffer, not only in day-to-day interactions but also in their capacity to cultivate a positive family environment for their offspring.

Supporting this, a study by Johnson et al. (2016) found that when male partners perceived their contributions to housework as fair, couples reported more frequent sexual encounters and greater sexual satisfaction. Ideally then, both partners would score highly on such traits, but as a fallback, having at least one highly family oriented partner may serve as a compensatory buffer, contributing to overall relationship quality.

This brings us to the next question: why do people prefer their partners to score higher than themselves on key mate value traits? The short answer is that they desire partners who are more desirable than themselves. This preference makes sense from both an evolutionary and an economic perspective. From an evolutionary standpoint, securing a partner with superior traits may increase reproductive success and social standing. Economically, it resembles a favorable exchange, much like bartering something of modest value for something more valuable, leading to a sense of gain and satisfaction. Similarly, in a business analogy, if one invests less while the partner contributes more, yet both share equal ownership, the arrangement is clearly advantageous from one’s own perspective. The same logic applies here: individuals who perceive their partners as possessing higher levels of the five mate value traits tend to report greater relationship satisfaction.

Homogamy, the preference for similar mates, is common across cultures. In modern China, homogamy and hypogamy (marrying someone with a lower education level) are linked to lower marriage satisfaction among women, but not men (Wang & Ma, 2025). As women’s education levels rise in China, marriage patterns, gender roles, and mating preferences are likely evolving. Unlike Norway, China has more traditional gender roles, greater economic inequality, and lacks a welfare state, all of which may influence mating preferences differently across countries.

A society similar to Norway, Finland, showed mixed and contradictory evidence for the likelihood of marriage among cohabiting couples, based on their socioeconomic and educational homogamy (Mäenpää & Jalovaara, 2013). The associations depended on the group and educational level—i.e., no evidence for the general impact of homogamy was found.

In the 20th century United States, educational homogamy fluctuated with socioeconomic inequality (Mare, 2016). Spousal similarity was higher in the early and late 20th century when economic inequality was greater, and lower around and after the mid-century when inequality was reduced.

We have previously established in our research that Norwegian mating preference may exhibit lower sex differences in some preferences (Mehmetoglu & Määttänen, 2020; Mehmetoglu et al., 2024). The aforementioned findings from China, Finland, and the US suggest that the impact of homogamy on pair-bonding and relationship satisfaction is influenced by culture, time period, socioeconomic development, and societal structures like the welfare state. Future research should include repeated measures across various countries and time points to better understand how mate similarity and homogamy affect pair-bonding, relationship satisfaction, and stability in different environments.

As implied earlier, family orientation emerges as the most important mate value trait in the Norwegian context. This is evident in both Figure 2 and Figure 3, where we observe that, unlike the other mate value traits, relationship satisfaction drops sharply when both partners score low on family orientation. In the other dimensions, satisfaction remains relatively low but still within an acceptable range, even when both partners have low scores.

As family orientation (i.e. understanding, kind, loyal, caring, family man/woman) of both partners was the most important predictor for relationship satisfaction in this study, it may suggest that features related to the relationship itself, rather than, say, materialistic preferences, are more important in egalitarian, wealthy welfare societies like Norway. This may also reflect the “collapse” of the extended family as the most important unit of raising children over the nuclear family, i.e., the couple itself. In the modern environment, the nuclear family dynamics, where both parents have the pressure to work outside the home, may lead to even stronger emphasis on the relationship itself and burden-sharing within the relationship. In egalitarian Norway (The Lancet Regional Health–Europe, 2025), family orientation is highly valued because there is a strong cultural expectation that both parents will contribute equally to household responsibilities and the upbringing of children. Moreover, family orientation is seen as an existential trait that children are expected to learn and internalize from an early age. As such, it plays a central role in shaping the expectations and norms of future relationships. A lack of family orientation is therefore unlikely to be viewed as advantageous in the context of serious or long-term relationships.

Norway is a wealthy and egalitarian country in terms of economic distribution and gender roles. This may impact also the mating preferences. According to “gender equality paradox”, more egalitarian societies would paradoxically have larger gender differences. On the other hand, our previous results suggest that in egalitarian Norway, the sex differences in mating preferences are either smaller or similar to populations with lower equality (Mehmetoglu & Määttänen, 2020; Mehmetoglu et al., 2024).

Interestingly, among the five mate value dimensions, physical condition—i.e., health and physical fitness—was the least sensitive to imbalances between partners’ scores, whether low or high. This suggests that, unlike other traits, physical condition may carry less direct weight in shaping relationship satisfaction. In other words, mismatches in this domain do not appear to significantly disrupt relational dynamics or have notable implications for offspring-related outcomes. One possible explanation is that, in the context of long-term relationships, which characterized the sample in this study, physical condition plays a less central role than it might during the initial stages of partner selection.

From a practical or clinical standpoint, the findings of this study offer meaningful insights for couple therapists. Specifically, mate value discrepancy can serve as a valuable framework for assessing and improving relationship dynamics. Therapists should consider incorporating this perspective into their practice if they have not already done so.

For example, in a hypothetical case where a woman expresses dissatisfaction with her relationship, the therapist could explore whether a significant discrepancy exists between her and her partner’s mate value, particularly in traits such as family orientation. It may be that she perceives herself as highly committed to family life, while her partner scores considerably lower on this trait. By making the partner aware of this imbalance, the therapist can encourage targeted behavioral changes—such as increasing involvement in household responsibilities or parenting—which may help reduce the discrepancy and enhance relationship satisfaction.

### Limitations and Future Research

Future studies should investigate how mate value discrepancy across specific dimensions, such as family orientation, appearance, sociability, physical condition and resourcefulness, relates not only to relationship satisfaction but also to negative outcomes such as conflict, instability, infidelity, or divorce. A further refinement would be to expand the set of mate value dimensions to include additional traits that may capture other salient features, particularly those that are context dependent.

A promising direction for future research involves the development of a structural equation model (SEM) that incorporates mate value discrepancy alongside potential mediating and moderating variables—such as personality, attitudes, values, or educational level—to better understand the mechanisms underlying its effects on relationship quality. Regarding moderators, the current study did not examine gender as a potential moderating factor. This decision was based on the assumption that, regardless of gender, individuals are likely to prefer partners with higher mate value traits in order to enhance the well-being and future prospects of their offspring. Nevertheless, this assumption remains empirical and should be tested in future research to explore whether gender moderates the relationship between mate value discrepancy and relationship outcome variables.

Another important limitation of our work is that self and partner assessments of mate value dimensions were based on perceived rather than objectively measured traits. Such perceptions are susceptible to cognitive biases, including idealization or devaluation of self and partner, projection, and relationship-based distortions. These biases may influence both the size of the measured discrepancies and relationship satisfaction itself. While perceived mate value remains a theoretically relevant construct, it should not be equated with actual traits. We recommend that readers keep this limitation in mind and that future studies complement subjective ratings with objective or externally verified indicators.

Finally, the current study was conducted in a highly egalitarian and gender-equal cultural context (Norway). Replicating this research in less egalitarian societies would offer valuable comparative insights. In such settings, one might hypothesize that scenarios where the partner scores higher than the self on key mate value traits (partner > self) may be especially conducive to greater relationship satisfaction due to traditional gender role expectations and asymmetrical power dynamics.

## Figures and Tables

**Table 1:** Descriptive overview of the sample characteristics.

Variable	Level	Count	Percent	*Mean*	*SD*	Min	Max
age		904		51.87	15.76	18	86
Relationship length	<3 months	1	0.1				
	3–6 months	1	0.1				
	6–12 month	8	0.9				
	1–2 years	20	2.2				
	2–4 years	64	7.1				
	4–6 years	53	5.9				
	6–10 years	75	8.3				
	10–20 years	207	22.9				
	>20 years	475	52.5				
gender	men	488	54				
	women	416	46				
Number of children under 18	none	622	68.8				
	1 kid	92	10.2				
	2 kids	128	14.2				
	3 kids	51	5.6				
	4 kids	9	1				
	>5 kids	1	0.1				
	do not want to answer	1	0.1				
Educational level	Secondary	36	4				
	High School	261	28.9				
	University 1–3 yrs	302	33.4				
	University 4 yrs +	224	24.8				
	University 5 yrs +	56	6.2				
	Other	25	2.8				

**Table 2:** Factor structure (standardized loadings). *M* = mean, λ = loading, SE = standard error, Z = z-value, α = Cronbach’s α.

Latent Variable	Label	*M*	λ	SE	Z	α
family orientation partner (Famp)	Understanding	3.93	0.80	0.01	57.93	0.86
	Kind	4.25	0.84	0.01	70.78	
	Loyal	4.29	0.80	0.01	57.47	
	Caring	4.22	0.83	0.01	66.85	
	Family man/woman	4.14	0.67	0.02	34.76	
resourcefulness partner (Resp)	Independent	3.80	0.56	0.02	22.55	0.81
	Intelligent	4.00	0.69	0.02	36.24	
	Successful	3.71	0.84	0.01	69.07	
	Looked up to	3.69	0.77	0.02	50.14	
	Has or likely to have good finance	3.63	0.63	0.02	30.57	
	Resourceful person	3.91	0.80	0.01	56.92	
appearance partner (Appp)	Attractive face	3.82	0.79	0.02	52.50	0.86
	Attractive body	3.50	0.87	0.01	74.69	
	Sexy	3.39	0.87	0.01	75.47	
sociability partner (Socp)	Humorous	3.56	0.83	0.01	56.98	0.81
	Social intelligent	3.83	0.68	0.02	32.75	
	Outgoing	3.69	0.63	0.02	27.55	
	Makes others laugh	3.54	0.82	0.01	56.37	
physical condition partner (Phyp)	Healthy	3.61	0.79	0.02	37.81	0.68
	In good physical condition	3.29	0.75	0.02	34.81	
family orientation nself (Fams)	Understanding	3.96	0.78	0.02	47.43	0.84
	Kind	4.12	0.79	0.02	48.93	
	Loyal	4.27	0.72	0.02	38.71	
	Caring	4.15	0.78	0.02	48.33	
	Family man/woman	4.00	0.52	0.03	19.99	
resourcefulness self (Ress)	Independent	3.84	0.51	0.03	18.64	0.82
	Intelligent	3.82	0.63	0.02	27.45	
	Successful	3.41	0.79	0.02	48.98	
	Looked up to	3.14	0.70	0.02	35.77	
	Has or likely to have good finance	3.48	0.58	0.02	25.27	
	Resourceful person	3.79	0.75	0.02	42.60	
appearance self (Apps)	Attractive face	3.01	0.76	0.02	43.68	0.85
	Attractive body	2.73	0.83	0.01	55.52	
	Sexy	2.44	0.82	0.02	53.76	
sociability self (Socs)	Humorous	3.32	0.82	0.02	52.87	0.81
	Social intelligent	3.64	0.61	0.02	25.68	
	Outgoing	3.45	0.58	0.02	23.69	
	Makes others laugh	3.42	0.87	0.01	61.64	
physical condition self (Phys)	Healthy	3.64	0.70	0.03	27.31	0.69
	In good physical condition	3.19	0.75	0.03	29.95	
relationship satisfaction (Rsat)	Your partner meets your needs	4.04	0.88	0.01	102.05	0.89
	You are satisfied with your relationship	4.20	0.93	0.01	143.85	
	Your relationship is good compared to most	4.12	0.84	0.01	77.32	
	You think that you should not have gotten into this relationship (reversed)	4.33	0.61	0.02	28.28	
	Your relationship has met your original expectations	3.77	0.64	0.02	30.47	
	You love your partner	4.38	0.77	0.01	53.52	
	You have problems in your relationship (reversed)	3.65	0.54	0.02	21.94	

## Data Availability

The raw data supporting the conclusions of this article will be made available by the authors on request.

## References

[B1-behavsci-15-01131] Barranti M., Carlson E. N., Côté S. (2017). How to test questions about similarity in personality and social psychology research: Description and empirical demonstration of response surface analysis. Social Psychological and Personality Science.

[B2-behavsci-15-01131] Bhogal M. S., Rhead C., Tudor C. (2019). Understanding digital dating abuse from an evolutionary perspective: Further evidence for the role of mate value discrepancy. Personality and Individual Differences.

[B3-behavsci-15-01131] Buss D. M., Goetz C., Duntley J. D., Asao K., Conroy-Beam D. (2017). The mate switching hypothesis. Personality and Individual Differences.

[B4-behavsci-15-01131] Buyukcan-Tetik A., Campbell L., Finkenauer C., Karremans J. C., Kappen G. (2017). Ideal standards, acceptance, and relationship satisfaction: Latitudes of differential effects. Frontiers in Psychology.

[B5-behavsci-15-01131] Conroy-Beam D., Goetz C. D., Buss D. M. (2016). What predicts romantic relationship satisfaction and mate retention intensity: Mate preference fulfillment or mate value discrepancies?. Evolution and Human Behavior.

[B6-behavsci-15-01131] Danel D. P., Siennicka A., Glińska K., Fedurek P., Nowak-Szczepańska N., Jankowska E. A., Lewandowski Z. (2017). Female perception of a partner’s mate value discrepancy and controlling behaviour in romantic relationships. Acta Ethologica.

[B7-behavsci-15-01131] Gyuris P., Járai R., Bereczkei T. (2010). The effect of childhood experiences on mate choice in personality traits: Homogamy and sexual imprinting. Personality and Individual Differences.

[B8-behavsci-15-01131] Hendrick S. S. (1988). A generic measure of relationship satisfaction. Journal of Marriage and the Family.

[B9-behavsci-15-01131] Howie J. M., Pomiankowski A., Weekes-Shackelford V. A., Shackelford T. K. (2021). Mate value. Encyclopedia of evolutionary psychological science.

[B10-behavsci-15-01131] Johnson M. D., Galambos N. L., Anderson J. R. (2016). Skip the dishes? Not so fast! Sex and housework revisited. Journal of Family Psychology.

[B11-behavsci-15-01131] Kirsner B. R., Figueredo A. J., Jacobs W. J. (2003). Self, friends, and lovers: Structural relations among beck depression inventory scores and perceived mate values. Journal of Affective Disorders.

[B12-behavsci-15-01131] Mare R. D. (2016). Educational homogamy in two gilded ages: Evidence from intergenerational social mobility data. The ANNALS of the American Academy of Political and Social Science.

[B13-behavsci-15-01131] Mäenpää E., Jalovaara M. (2013). The effects of homogamy in socio-economic background and education on the transition from cohabitation to marriage. Acta Sociologica.

[B14-behavsci-15-01131] Mehmetoglu M., Määttänen I. (2020). Norwegian men and women value similar mate traits in short-term relationships. Evolutionary Psychology.

[B15-behavsci-15-01131] Mehmetoglu M., Määttänen I., Mittner M. (2024). Testing sexual strategy theory in norway. Behavioral Sciences.

[B16-behavsci-15-01131] Rosseel Y. (2012). lavaan: An R package for structural equation modeling. Journal of Statistical Software.

[B17-behavsci-15-01131] Schacht R., Kramer K. L. (2019). Are we monogamous? A review of the evolution of pair-bonding in humans and its contemporary variation cross-culturally. Frontiers in Ecology and Evolution.

[B18-behavsci-15-01131] Schönbrodt F. D., Humberg S. (2023). RSA: An r package for response surface analysis (version 0.10.6).

[B19-behavsci-15-01131] Shanock L. R., Baran B. E., Gentry W. A., Pattison S. C., Heggestad E. D. (2010). Polynomial regression with response surface analysis: A powerful approach for examining moderation and overcoming limitations of difference scores. Journal of Business and Psychology.

[B20-behavsci-15-01131] Sidelinger R. J., Booth-Butterfield M. (2007). Mate value discrepancy as predictor of forgiveness and jealousy in romantic relationships. Communication Quarterly.

[B21-behavsci-15-01131] Singh Bhogal M. S., Howman J. M. (2019). Mate value discrepancy and attachment anxiety predict the perpetration of digital dating abuse. Evolutionary Psychological Science.

[B22-behavsci-15-01131] Starratt V. G., Weekes-Shackelford V. A., Shackelford T. K. (2017). Mate value both positively and negatively predicts intentions to commit an infidelity. Personality and Individual Differences.

[B23-behavsci-15-01131] Štěrbová Z., Třebický V., Havlíček J., Tureček P., Correa Varella M. A., Varella Valentova J. (2018). Father’s physique influences mate preferences but not the actual choice of male somatotype in heterosexual women and homosexual men. Evolution and Human Behavior.

[B24-behavsci-15-01131] Štěrbová Z., Tureček P., Kleisner K. (2019). Consistency of mate choice in eye and hair colour: Testing possible mechanisms. Evolution and Human Behavior.

[B25-behavsci-15-01131] The Lancet Regional Health–Europe (2025). The paradox of prosperity and poverty: Confronting inequality in norway. The Lancet Regional Health-Europe.

[B26-behavsci-15-01131] Thiessen D., Gregg B. (1980). Human assortative mating and genetic equilibrium: An evolutionary perspective. Ethology and Sociobiology.

[B27-behavsci-15-01131] Wang X., Ma C. (2025). Educational assortative mating and its impact on marital quality in china. Journal of Family Issues.

[B28-behavsci-15-01131] Young G., Zeigler-Hill V., Ross J. (2024). Mate value discrepancies and mate retention behaviors: A cubic response surface analysis. Personal Relationships.

